# Physicochemical Characterization, Prebiotic Potential, and Lipid-Lowering Effect of *Mesembryanthemum crystallinum* L. Polysaccharide

**DOI:** 10.3390/foods15071153

**Published:** 2026-03-27

**Authors:** Hui Cao, Bing Yang, Yangyang Wang, Jingjing Zhang, Huaxing Xiong, Haolin Zhang, Zhanhui Cao, Hui Teng, Lei Chen, Hui Wang

**Affiliations:** 1 College of Food Science and Technology, Guangdong Ocean University, Guangdong Provincial Key Laboratory of Aquatic Product Processing and Safety, Guangdong Province Engineering Laboratory for Marine Biological Products, Guangdong Provincial Engineering Technology Research Center of Seafood, Guangdong Provincial Engineering Technology Research Center of Prefabricated Seafood Processing and Quality Control, Zhanjiang 524088, Chinabingyang_1268@163.com (B.Y.);; 2 Collaborative Innovation Center of Seafood Deep Processing, Dalian Polytechnic University, Dalian 116034, China; 3 Nutrition and Bromatology Group, Department of Analytical and Food Chemistry, Faculty of Science, Universidade de Vigo, 32004 Ourense, Spain

**Keywords:** *Mesembryanthemum crystallinum* L., polysaccharide, gut microbiota, metabolomics, obesity

## Abstract

Excessive lipid accumulation, a hallmark characteristic of high-fat diet (HFD)-induced obesity, has become a worldwide challenge, necessitating the exploration of secure and efficacious natural products for its intervention. In the present work, a polysaccharide (MCP) was extracted and purified from *Mesembryanthemum crystallinum* L., a novel halophyte, and its physicochemical properties, in vitro fermentation characteristics, lipid-lowering activity, and underlying mechanisms were systematically investigated. Physicochemical analysis revealed that MCP is an acidic polysaccharide, with galacturonic acid as the predominant monosaccharide component, broad molecular weight distribution, and a porous structural morphology. In vitro fermentation experiments demonstrated that MCP could be effectively utilized by human fecal microbiota, significantly promoting the yield of short-chain fatty acids (SCFAs), particularly butyrate at high concentrations, which outperformed inulin. 16S rDNA sequencing uncovered that MCP optimized microbiota composition by enriching SCFA-producing beneficial bacteria (*Prevotella_9*, *Faecalibacterium*) while suppressing opportunistic pathogens (*Megamonas*, *Escherichia-Shigella*). Metabolomic analysis of fermentation broth revealed that MCP significantly affected microbial glycerophospholipid metabolic pathways. Experiments in *Caenorhabditis elegans* (*C. elegans*) confirmed that MCP inhibited HFD-induced lipogenesis, which was linked to the regulation of the *nhr-49*/*sbp-1*-mediated lipogenesis pathway. For the first time, using an antibiotic-induced microbiota depletion model in *C. elegans*, the lipid-lowering effect of MCP was observed to disappear, suggesting a potential role of the gut microbiota in mediating this effect. This investigation establishes a scientific basis for MCP as a novel prebiotic or dietary supplement for managing obesity-related lipid accumulation.

## 1. Introduction

In 2020, approximately 42% of adults worldwide were classified as overweight (body mass index (BMI) ≥ 25 kg/m^2^) or obese (BMI ≥ 30 kg/m^2^), according to the World Obesity Federation, a figure projected to reach 50% by 2030 [1]. Beyond its classification as excess body weight, this condition is recognized as a central driver of metabolic syndrome, a pathological cluster encompassing insulin resistance, dyslipidemia, and hypertension [2]. Chronic energy surplus disrupts systemic metabolic equilibrium, particularly impairing lipid homeostasis [3]. Such dysregulation enhances lipid deposition while suppressing fatty acid oxidation and thermogenic processes [4], thereby predisposing individuals to life-threatening conditions including cardiovascular disease, diabetes, skeletal disorders, and certain cancers [5]. Consequently, in 2021, high BMI was attributable to an estimated 3.7 million deaths, underscoring its role as a contributor to global mortality and morbidity [6]. Given the protracted nature of obesity management, exploring natural bioactive compounds with lipid-lowering or anti-obesity properties is recognized as a safe and highly viable strategy [7].

Growing evidence has established a robust correlation between gut microbes and host metabolic regulation [8]. In obesity, consistent compositional and functional shifts in the gut microbial community—typically marked by an elevated Firmicutes/Bacteroidetes ratio and diminished microbial diversity—have been well documented [9]. This dysbiosis actively drives the progression of obesity and its associated metabolic disorders through diverse mechanistic pathways. For example, gut microbiota-derived short-chain fatty acids (SCFAs) can modulate lipid metabolism, energy expenditure, and insulin sensitivity [10]. Furthermore, microbiota-mediated alterations in bile acid metabolism can disrupt lipid homeostasis [11]. These insights have positioned the modulation of gut microbiota composition and function as a promising therapeutic strategy for obesity management.

Plant polysaccharides are high molecular weight polymers comprising ten or more monosaccharide units linked by glycosidic bonds [12]. Undigested in the upper gastrointestinal tract, non-starch polysaccharides travel to the colon intact and are utilized as substrates by gut bacteria. Through this prebiotic effect, polysaccharides are capable of reshaping the microbial community and metabolic output, thereby influencing host energy metabolism [13]. Research on plant polysaccharides for obesity mitigation has primarily focused on their ability to regulate lipid metabolism and modulate gut microbiota, with emerging evidence also implicating improvements in intestinal barrier function and inflammation [14,15,16].

*Mesembryanthemum crystallinum* L. (*M. crystallinum* L.) is a succulent perennial halophyte belonging to the family Aizoaceae, widely distributed in coastal regions [17]. Indigenous to eastern and southern Africa, this species is now cultivated in China and globally [18]. *M. crystallinum* L. adapts to extreme environments by synthesizing protective substances and antioxidant molecules in its stems and leaves, which contain specialized structures called vacuolar cells [19]. Traditionally valued for its edibility and medicinal properties, it has been used to clear heat, promote diuresis, and relieve asthma. Studies confirm its richness in bioactive compounds and natural mineral elements, thereby exhibiting potential antioxidant and anti-inflammatory activities with possible advantages for conditions such as hypertension and hyperglycemia [20]. Polysaccharides extracted from *M. crystallinum* by M’sakni et al. consist primarily of highly methylesterified homogalacturonan (partially linked to non-methylesterified HG) and two distinct types of rhamnogalacturonan-I [21]. However, the activity of *M. crystallinum* polysaccharides in alleviating obesity has not yet been investigated.

Therefore, the present study hypothesized that polysaccharides extracted from *M. crystallinum* L. may exert beneficial effects on lipid metabolism. To test this hypothesis, this study first characterized *M. crystallinum* L. polysaccharides (MCP) extracted via a hot water method and evaluated their prebiotic effects on intestinal microbiota and associated metabolic pathways during in vitro fermentation. Additionally, the hypolipidemic activity of MCP was assessed utilizing a high-fat diet (HFD)-driven lipid accumulation model in *Caenorhabditis elegans* (*C. elegans*), which reflects lipid accumulation under dietary stress. This study provides initial insights into the potential of MCP in modulating lipid metabolism, thereby laying a foundation for future investigations into its value as a health-promoting food ingredient.

## 2. Materials and Methods

### 2.1. Materials and Reagents

*M. crystallinum* L. was sourced from Weifang, Shandong, China. An assay kit for total triglycerides (TG) was sourced from Nanjing Jiancheng Institute (Nanjing, China). FreeZol Reagent was procured from Vazyme Biotech Co., Ltd. (Nanjing, China). The Evo M-MLV RT Mix Kit with gDNA Clean for qPCR Ver.2 and SYBR Green Premix Pro Taq HS qPCR Kit were supplied by Accurate Biology (Changsha, China). Sigma-Aldrich (St. Louis, MO, USA) was the commercial source for all monosaccharide standards used in this study, namely Fuc, Rha, Ara, Gal, Glc, Xyl, Man, Fru, Rib, Gal-UA, Gul-UA, Glc-UA, and Man-UA. The remaining reagents were of analytical purity or chromatographic purity.

### 2.2. Extraction and Chemical Composition Analysis of MCP

#### 2.2.1. Extraction of MCP

The isolation of MCP followed the protocol of Ye et al. [22], incorporating minor changes. *M. crystallinum* L. was dried to a constant weight in a 55 °C oven and then ground into powder using a pulveriser. The powder was suspended in 80% ethanol in a proportion of 1:20 and stirred at 55 °C (30 min). The precipitate was dried completely at 95 °C. It was subsequently combined with water in a 1:30 proportion and extracted with stirring at 95 °C for 1.5 h. The supernatant was collected and concentrated to one-fifth of the initial volume. Proteins were isolated from the solution following the Sevag method. Then, anhydrous ethanol was added to the solution to reach a final concentration of 80% (*v*/*v*), and the mixture was left to settle for 14 h. The alcohol-precipitated polysaccharide was collected, redissolved, and dialysed for 48 h using an 8000–14,000 Da dialysis bag. Ultimately, the solution was lyophilized to yield *M. crystallinum* L. polysaccharide (MCP). Ash content was determined following the protocol outlined by Song et al. [23].

#### 2.2.2. Assessment of Neutral Sugar Content

The phenol–sulfuric acid assay was employed with glucose as the standard (0–0.05 mg/mL) [24]. 500 μL of sample or standard was mixed with 500 μL 5% phenol and 2.5 mL sulfuric acid, allowed to react for 20 min and measured at 490 nm.

#### 2.2.3. Assessment of Uronic Acid Content

The carbazole–sulfuric acid assay was employed with galacturonic acid as the reference (0–0.10 mg/mL) [25]. Under ice-cold conditions, 250 µL of sample or standard was blended with 1.5 mL of sodium tetraborate–sulfuric acid solution, then kept at 85 °C (20 min). After cooling, 50 μL 0.1 mg/mL carbazole in ethanol was added, allowed to stand for 2 h and measured at 530 nm.

#### 2.2.4. Assessment of Protein Content

The Bradford assay was employed with bovine serum albumin (BSA) as the standard (0–0.10 mg/mL) [26]. 1.5 mL of sample or standard was mixed with 5.0 mL of Bradford reagent, incubated at room temperature for 5 min, and the absorbance was measured at 595 nm.

#### 2.2.5. Assessment of Total Polyphenol Content

The Folin–Ciocalteu assay was employed with gallic acid as the reference (0–0.5 mg/mL) [27]. 80 μL of sample or standard was sequentially mixed with 320 μL of ultrapure water and 400 μL of 1 mol/L Folin–Ciocalteu reagent. After standing for 5 min, 400 μL of 20% Na_2_CO_3_ and 3.8 mL ultrapure water were added, allowed to incubate for 1 h, and measured at 760 nm.

#### 2.2.6. Assessment of Total Flavonoid Content

The aluminum nitrate colorimetric assay was employed with rutin as the standard (0–0.5 mg/mL) [28]. 200 μL of sample or standard was sequentially mixed with 400 μL of 0.066 mol/L NaNO_2_ (standing for 5 min). Then, 60 μL of 10% Al(NO_3_)_3_ was added and left for 5 min, followed by the addition of 400 μL of 1 mol/L NaOH. After a 15 min reaction at room temperature, the absorbance was measured at 510 nm.

### 2.3. Characterisation of MCP

#### 2.3.1. Assessment of Molecular Weight

MCP was prepared at 1 mg/mL in 0.1 M NaNO_3_ containing 0.02% NaN_3_ and passed through a 0.45 µm filter. SEC-MALLS-RI was employed to assess the homogeneity and molecular mass of each fraction [29]. Using a DAWN HELEOS-II laser photometer (Wyatt Technology, Santa Barbara, CA, USA) fitted with tandem Shodex OH-pak SB-805 and 803 columns (300 × 8 mm, Showa Denko, Tokyo, Japan), the weight-average (Mw) and number-average (Mn) molecular weights were determined. Column temperature was maintained at 45 °C with a Sanshu Biotech heater (Shanghai, China), and the mobile phase flowed at 0.6 mL/min. An Optilab T-rex differential refractive index detector (Wyatt Technology, Santa Barbara, CA, USA) was connected inline to measure fraction concentration and dn/dc value, which was calculated as 0.141 mL/g in the same NaNO_3_/NaN_3_ solvent.

#### 2.3.2. Monosaccharide Composition Analyses

A 5 mg aliquot of MCP was subjected to hydrolysis in a sealed tube with 2 M trifluoroacetic acid at 121 °C for 2 h [30]. Following hydrolysis, the mixture was evaporated under a nitrogen stream. The residue was then rinsed with methanol and evaporated again (2–3 times). The material was taken up in deionized water and passed through a 0.22 µm filter. Monosaccharide composition was determined using HPAEC with a CarboPac PA-20 column (3 × 150 mm) coupled with a pulsed amperometric detector (PAD, Dionex ICS 5000+, Thermo Fisher Scientific, Waltham, MA, USA). Separation was performed using a ternary mobile phase: (A) ddH_2_O, (B) 0.1 M NaOH, and (C) 0.1 M NaOH + 0.2 M NaAc, at a flow rate of 0.5 mL/min with a 5 µL injection volume. The gradient was set as follows: 0 min (95% A, 5% B, 0% C); 26 min (85% A, 5% B, 10% C); 42 min (85% A, 5% B, 10% C); 42.1 min (60% A, 0% B, 40% C); 52 min (60% A, 40% B, 0% C); 52.1–60 min (95% A, 5% B, 0% C). The standards, standard curves, and correlation coefficients (R^2^) used for monosaccharide determination were presented in Table 1.

#### 2.3.3. UV and FT-IR Spectra Analysis

MCP (1 mg/mL) was analyzed by UV-Vis spectroscopy (Agilent, Santa Clara, CA, USA) across the 200–400 nm range [31]. This scanning was done to identify the ultraviolet absorption characteristics of proteins and nucleic acids present in the solution. This analysis was undertaken to determine whether these biomolecules were present within the MCP.

The functional groups of MCP were characterised using Fourier Transform Infrared (FTIR) spectroscopy [32]. In brief, MCP was blended with KBr at a ratio of 1:150, ground into powder, and pressed into 1 mm thick pellets. These were then analysed using a TENSOR 27 FT-IR spectrometer (Bruker, Ettlingen, Germany) within 4000–400 cm^−1^ spectral range.

#### 2.3.4. SEM and AFM Analyses

MCP was fixed onto conductive adhesive and gold-sputtered using a Quorum SC7620 sputter coater at 10 mA [33]. Subsequently, the morphological features of MCP were observed at various magnifications using a TESCAN MIRA LMS scanning electron microscope (TESCAN, Brno, Czech Republic). The detector employed was an SE2 secondary electron detector. The images were acquired at a resolution of 2048 × 1894 pixels and saved in TIFF format. The working distance was 15.22 mm. Quantitative analysis was conducted using ImageJ 1.8.0 software [34].

The 0.05 mg/mL MCP solution was prepared. A minute volume of the solution under consideration was dispensed onto a mica sheet and dried at room temperature [35]. Subsequent testing was conducted using a Bruker Dimension Icon AFM instrument (Bruker, Berlin, Germany). The probe employed was of the tapping mode variety. The images were acquired at a resolution of 1320 × 1080 pixels and saved in TIFF format.

### 2.4. In Vitro Microbiota Fermentation

#### 2.4.1. Faecal Bacterial Suspension

Faecal bacterial suspension was prepared following the method described by Zhang et al. with minor modifications [36]. Feces were gathered from six healthy participants (3 males and 3 females, 20–25 years old) who had not experienced intestinal disorders or undergone antibiotic treatment within the preceding three months. Each sample was transferred into a 50 mL sterile tube filled with 30 mL PBS (0.1 M, pH 7.0) and 30 glass beads. The weight before and after sampling was recorded to calculate the concentration (g/mL). After vortex homogenisation, the mixture was filtered through three layers of sterile gauze. The supernatant from each tube was diluted to 0.1 g/mL. Diluted faecal supernatants were mixed 1:1 (*v*/*v*) for subsequent inoculation.

Informed consent was obtained from all donors. The sample collection procedure involved in this study did not pose any physical, psychological, legal, or informational risks to the participants. In this study, the faecal microbiota was only used as a medium for polysaccharide fermentation, and the research content did not involve biomedical research directly related to human health. This study commenced in January 2025. According to the “Measures for Ethical Review of Life Science and Medical Research Involving Human Being” (National Health Commission of the People’s Republic of China, 2023), Article 32, research using anonymized human biological samples may be exempted from ethics review under certain conditions. All fecal samples were anonymized prior to use, and no identifiable personal information was collected or accessed.

#### 2.4.2. Preparation of Culture Media

The medium was prepared as described [37]. Medium components: 10.0 g/L trypticase peptone, 2.5 g/L yeast extract, 0.09 g/L MgSO_4_·7H_2_O, 0.09 g/L CaCl_2_, 0.45 g/L KH_2_PO_4_, 0.45 g/L K_2_HPO_4_, 0.9 g/L NaCl, 1.5 g/L NaHCO_3_, 1.0 g/L L-cysteine, 0.8 mg/L gentian violet solution, 10.0 mg/L haem, 5.0 mg/L vitamin B_2_, 10.0 mg/L vitamin B_6_, 2.0 mg/L vitamin B_7_, 0.1 mg/L vitamin B_12_, 2.0 mg/L folic acid and 5.0 mg/L para-aminobenzoic acid. The medium was adjusted to pH 7.20 and filter-sterilized through a 0.22 µm membrane.

#### 2.4.3. Fermentation System and Experimental Design

Inulin, a well-established prebiotic fiber, was used as a positive control to allow direct comparison [38]. Groups: Control group (CON); Positive group (INU) received Inulin (5 mg/mL); Low-dose MCP group (L_MCP) received MCP (2.5 mg/mL); High-dose MCP group (H_MCP) received MCP (5 mg/mL).

An anaerobic fermentation system was employed [39]. Fermentations were carried out in 5 mL anaerobic fermentation tubes with a working volume of 2.5 mL. Anaerobic conditions were maintained by flushing with nitrogen gas. Each tube contained 2.25 mL of sterile culture medium and was inoculated with 0.25 mL of the faecal bacterial suspension (10%, *v*/*v*). Six fermentation time points were established: 0, 3, 6, 12, 24, and 48 h. At each time point, three independent parallel samples were prepared for every experimental group. Fermentation tubes were incubated in a shaking incubator (shaking speed 100 rpm, temperature 37 °C).

#### 2.4.4. Assessment of pH and Carbohydrate Content

The fermentation broth was centrifuged at 10,000× *g* for 10 min at 4 °C. The pH of each sample was measured with a pH meter (Mettler-Toledo, Greifensee, Switzerland). The phenol-sulfuric acid assay, as described in Section 2.2, was used to evaluate residual carbohydrates.

#### 2.4.5. Assessment of SCFAs

SCFAs were analyzed by gas chromatography [40]. In brief, 250 µL of 15% phosphoric acid solution was blended with 250 µL of fermentation supernatant. Then, 1 mL ethyl acetate was blended. After centrifugation (12,000 rpm, 10 min, 4 °C), the supernatant was aspirated and passed through a 0.22 µm filter. Subsequently, the levels and composition of SCFAs were analysed using a GC-2030 system (Shimadzu, Kyoto, Japan) connected to a flame ionisation detector and an SH-Wax column. Both the detector and injector were maintained at 250 °C. The column temperature was initially kept at 60 °C. SCFA levels were calculated from target peak areas using standard calibration equations.

#### 2.4.6. 16S rDNA Sequencing-Fermentation Broth Microbial Community

Following fermentation, the fermentation broth was subjected to centrifugation (10,000× *g*, 10 min, 4 °C), and the pellet was obtained. DNA was isolated from each sample with a bacterial genomic extraction kit (TIANGEN, Beijing, China). The V3–V4 region was amplified by PCR via primers 341F/805R (341F: 5′-CCTACGGGNGGCWGCAG-3′, 805R: 5′-GACTACHVGGGTATCTAATCC-3′) [41]. After purification, PCR products were analyzed on an Agilent 2100 Bioanalyzer (Agilent, Santa Clara, CA, USA) and quantified with Illumina library quantification kits (Kapa Biosciences, Woburn, MA, USA). Libraries were then sequenced utilizing a NovaSeq 6000 sequencer.

#### 2.4.7. Determination of Metabolites in Fermentation Broth

Sample preparation for LC–MS (Vanquish UHPLC and Q Exactive HF-X MS, Thermo Scientific, Waltham, MA, USA) began by adding 150 µL of extraction solvent (acetonitrile:methanol = 1:4, *v*/*v*, with internal standard) to 50 µL of sample [42]. The mixture was vortexed for 3 min and centrifuged (12,000 rpm, 10 min, 4 °C). A 150 µL portion of the supernatant was then incubated at −20 °C (30 min), followed by a second centrifugation step (12,000 rpm, 3 min, 4 °C). The final supernatant (120 µL) was transferred to an autosampler vial for analysis. A Waters ACQUITY Premier HSS T3 column (1.8 µm, 2.1 × 100 mm) was used for separation. The mass spectrometer was operated in ESI positive and negative modes, scanning from *m*/*z* 75–1000 at 35,000 resolution. Operating parameters included: Ion spray voltage, 3.5 KV or 3.2 KV in positive or negative modes, respectively; Sheath gas (Arb), 30; Aux gas, 5; Ion transfer tube temperature, 320 °C; Vaporizer temperature, 300 °C; Collision energy, 30, 40, 50 V; Signal Intensity Threshold, 1.00 × 10^6^ cps; Top N vs. Top speed, 10; Dynamic exclusion, 3 s.

### 2.5. Lipid-Lowering Activity in C. elegans

#### 2.5.1. C. elegans Culture

*C. elegans* was cultivated using nematode growth medium (NGM) plates within a 20 °C constant-temperature incubator. The surface of the NGM plates was coated with an *E. coli* OP50 suspension, thus providing nutrients for the nematodes. The *C. elegans* employed in this study were wild-type (N2). A population of first-stage larvae (L1) synchronised to the same age was obtained through the bleaching of pregnant hermaphrodites with NaOH and HClO, followed by centrifugation to purify the eggs and subsequent overnight incubation in M9 buffer. A synchronised population of fourth-stage nematodes (L4) was harvested 48 h after L1 synchronisation for subsequent experiments.

#### 2.5.2. Construction of Lipid Accumulation Model

Oleic acid (OA) and palmitic acid (PA) were reported to induce lipid accumulation in *C. elegans* [43]. In this experiment, fatty acid supplementation employed a mixed solution with final concentrations of 1 mM OA and 1 mM PA. Synchronised L4 nematodes were randomly divided and cultured, respectively, under four treatment conditions containing 100 µM 5-fluoro-2′-deoxyuridine (FuDR) and inactivated OP50 bacteria: the control group (CON), high-fat diet (HFD), high-fat diet + low-concentration MCP (2 mg/mL MCP), and high-fat diet + high-concentration MCP (4 mg/mL MCP). After 6 days of incubation at 20 °C in a constant-temperature incubator, the nematodes (which had reached the Day 6 adult stage) from each group were collected using M9 buffer. At least three biological replicates were employed for all assays.

#### 2.5.3. Antibiotic Pretreatment

The L4-stage nematodes were exposed to 50 µg/mL gentamicin during the 12 h treatment, then randomly distributed and cultured, respectively, under three treatment conditions supplemented with 100 µM 5-fluoro-2′-deoxyuridine (FuDR) and inactivated OP50 bacteria: the control (CON), the high-fat diet (HFD), and the high-fat diet + high-concentration MCP (4 mg/mL MCP) group.

#### 2.5.4. Oil-Red-O Staining and Quantification

The Day 6 adult-stage nematodes were exposed to 4% paraformaldehyde (30 min). They were subjected to three freeze–thaw cycles, each consisting of freezing (2 min) and thawing in water (1 min). After fixation, these nematodes were washed in M9 buffer, incubated in 60% isopropanol for 10 min, and then stained with Oil Red O (ORO) working solution for 3 h with gentle agitation. Excess dye was removed by washing with 60% isopropanol, followed by three washes with M9 buffer. The nematodes were then placed on 2% agar plates and imaged using a B60F upright fluorescence biological microscope (Daoyi, Guangzhou, China) under brightfield settings. Images were captured for 40 nematodes per group using identical imaging parameters. ORO intensity, body length, and body width were measured across the whole body of each nematode using ImageJ 1.8.0 processing software.

#### 2.5.5. Quantification of ROS

The Day 6 adult-stage nematodes were mixed with 500 µL 10 µM 2,7-dichlorodihydrofluorescein-diacetate (H_2_DCF-DA) and shaken gently in the dark for 2.5 h. Subsequently, the nematodes were washed three times with M9 buffer and anesthetized with 500 µL of 1 mM levamisole. The nematodes were then placed on 2% agar plates and imaged using a B60F upright fluorescence biological microscope (Daoyi, Guangzhou, China) in dark-field configuration. 40 nematodes per group were photographed under identical imaging parameters. Fluorescence intensity was quantified across the whole body of each nematode using ImageJ 1.8.0 processing software.

#### 2.5.6. Triglyceride Determination

Triglyceride levels in the Day 6 adult-stage nematodes (approximately 2000 nematodes per group, 3 independent biological replicates) were determined using a triglyceride assay kit (Nanjing Jiancheng Bioengineering Institute, Nanjing, China) according to the manufacturer’s instructions.

#### 2.5.7. RT-qPCR Analysis

Total RNA was extracted from the Day 6 adult-stage nematodes (approximately 2000 nematodes per group, 3 independent biological replicates) using FreeZol Reagent (Vazyme Biotech Co., Ltd., Nanjing, China). RNA was then reverse transcribed into cDNA using the Evo M-MLV RT Mix Kit with gDNA Clean for qPCR Ver.2 (Accurate Biology, Changsha, China). The quantitative real-time qPCR (RT-qPCR) was undertaken for target genes utilising the SYBR Green Premix Pro Taq HS qPCR Kit (Accurate Biology, Changsha, China) and the fluorescent quantitative PCR system (Bio-Rad, Hercules, CA, USA). Target gene expression values were obtained using the 2^−ΔΔCt^ algorithm after normalization to the *act-1* reference gene.

### 2.6. Statistical Analysis

For microbiota sequencing data and non-targeted metabolomics data, detailed statistical procedures—including quality control, differential abundance analysis, multiple testing correction, and multivariate analysis—were described in Supplementary Methods. Each group contained 3 independent replicates (*n* = 3).

For the chemical composition analysis, all assays were performed in triplicate, and the results were expressed as mean ± SD. For other data, statistical analyses were performed using GraphPad 8.0.2 (GraphPad Software, San Diego, CA, USA) or SPSS 24 (SPSS Inc., Chicago, IL, USA). Normality of data distribution was assessed using the Shapiro–Wilk test. Homogeneity of variances was evaluated using Brown-Forsythe test. For datasets that met both normality and homoscedasticity assumptions, comparisons among multiple groups were performed using one-way analysis of variance (ANOVA) followed by Tukey’s test. For datasets that violated either assumption, the non-parametric Kruskal–Wallis test was used, followed by Dunn’s test for multiple comparisons. *p* < 0.05 was considered statistically significant in this study. Data represent mean ± SEM.

## 3. Results

### 3.1. Characterisation Analysis of MCP

Pigments and other substances were removed via ethanol extraction, followed by deproteinisation, alcohol precipitation, and dialysis to obtain MCP. The fresh *M. crystallinum* L. exhibited a dry matter content of 4.45 ± 0.04%, yielding polysaccharides at 4.05%. The chemical composition and contents of MCP were presented in Table 2. The neutral sugar content of MCP was 16.28 ± 0.23%. Meanwhile, the uronic acid content of MCP was 75.25 ± 3.89%. To assess the purity of the polysaccharide preparation, the levels of potential non-polysaccharide constituents were quantified. The measured contents of protein, total polyphenols, total flavonoids, and ash were 2.03 ± 1.07%, 0.16 ± 0.01%, 0.08 ± 0.04%, and 0.71 ± 0.16%, respectively.

**Figure 1:**
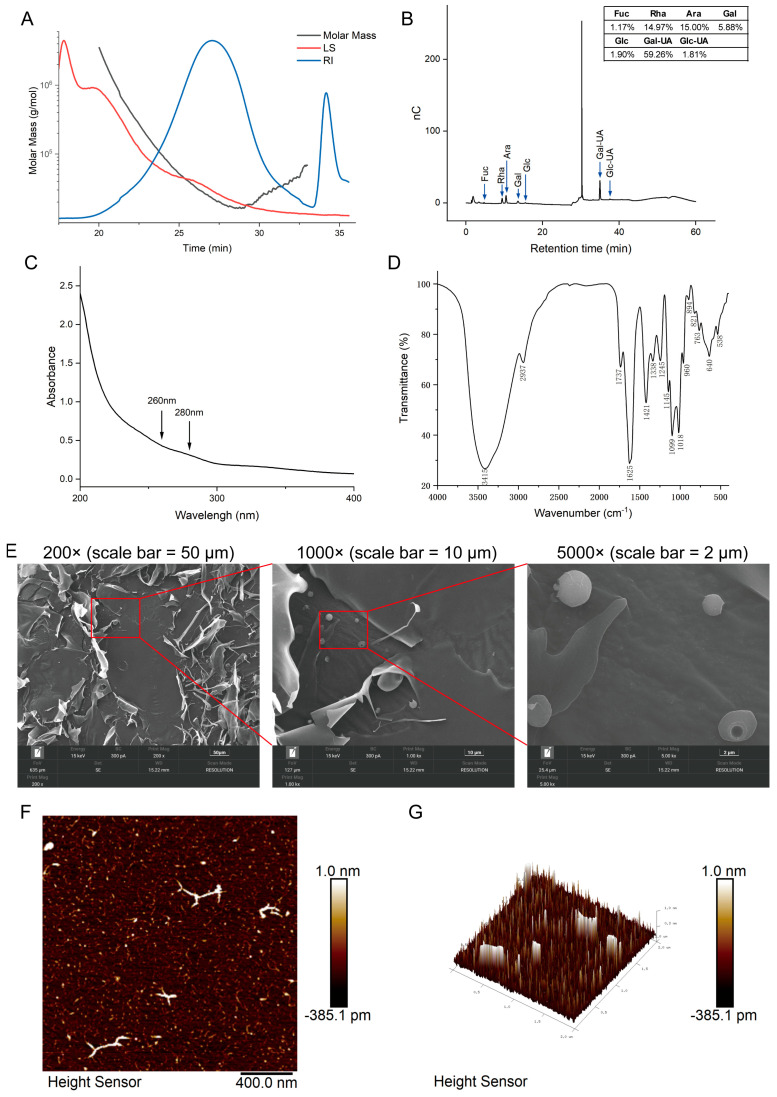
(**A**) Chromatogram of the molar mass distribution of MCP. (**B**) Monosaccharide composition profile. (**C**) UV absorption profile. (**D**) FT-IR analysis. (**E**) SEM images of MCP at magnifications of 200× (scale bar = 50 μm), 1000× (scale bar = 10 μm), and 5000× (scale bar = 2 μm). AFM scanning of MCP (scale bar = 400.0 nm): (**F**) two-dimensional imaging and (**G**) three-dimensional imaging.

Determination of MCP molecular weight was achieved by GPC-RI-MALS. Figure 1A showed that MCP comprised two major polysaccharide components: Component 1 (predominant, Mw 42.9 kDa, 89.07%) and Component 2 (Mw 9.7 kDa, 10.93%). The polydispersity index (PDI) was 2.632, indicating a broad molecular weight distribution.

The monosaccharide composition analysis of MCP (Figure 1B) revealed that MCP comprised Fuc, Rha, Ara, Gal, Glc, Gal-UA, and Glc-UA, accounting for 1.17%, 14.97%, 15.00%, 5.88%, 1.90%, 59.26%, and 1.81%, respectively. MCP contained 61.07% uronic acid.

UV-Vis scanning of MCP between 200 and 400 nm (Figure 1C) revealed no distinct absorbance at 260 or 280 nm, indicating that the preparation was essentially free of nucleic acids and proteins. The FT-IR spectrum of MCP exhibited characteristic features of polysaccharides (Figure 1D). The broad absorption at 3415 cm^−1^ was indicative of hydroxyl (O-H) stretching vibrations. A weak C-H stretching vibration was present at 2937 cm^−1^. Absorption peaks at 1737 cm^−1^ and 1625 cm^−1^ corresponded to the stretching vibrations of free carboxylic acid and carboxylate ions (-COO^−^). Peaks at 1421 cm^−1^ and 1338 cm^−1^ originated from C-H bending vibrations. Additionally, absorption peaks in the 1245–1018 cm^−1^ range (C-O-C and C-OH stretching) and at 960 cm^−1^ and 894 cm^−1^ (characteristic absorption of the β-glycosidic bond) further confirmed the polysaccharide structure.

At 200× magnification, MCP exhibited irregularly dispersed, curled flake-like and filamentous structures (Figure 1E). The area enclosed by the red box in 200× image is shown at higher magnification (1000×) in the middle panel, and the area enclosed by the red box in 1000× image is further magnified (5000×) in the right panel. With increasing magnification (1000×/5000×), spherical structures with surface-attached “perforations” were observed. Quantitative measurements of these spherical structures were provided in Figure S1. AFM two-dimensional imaging revealed randomly distributed irregular spots on the sample surface (Figure 1F). This phenomenon could be attributed to interactions between hydroxyl groups on polysaccharide chains, promoting the formation of highly intertwined structures. The height profile of MCP ranged from −385.1 pm to 1.0 nm. Typically, the height of a single polysaccharide chain was <1 nm. The broad height range observed in MCP (1.3851 nm total range) suggested the presence of polysaccharide components with varying molecular weights or branching degrees within the sample. AFM three-dimensional imaging (Figure 1G) showed rod-like molecular aggregates intertwining to form irregular aggregates, thereby confirming the locally aggregated, chain-entangled structure of MCP.

### 3.2. Analysis of pH, MCP Consumption and SCFAs

**Figure 2:**
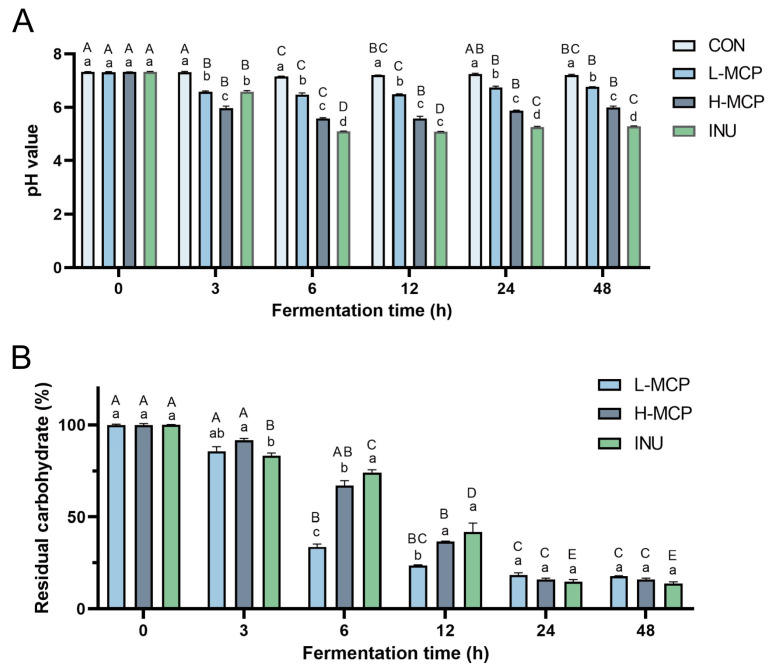
Changes in pH (**A**) and residual carbohydrate (**B**) during MCP fermentation. Data represent mean ± SEM (*n* = 3); normalized to 100% at 0 h. Lowercase letters denote significant differences between groups at a given time point, whereas uppercase letters indicate significant changes within the same group over time.

As shown in Figure 2A, the initial pH values of the MCP and INU groups were comparable to the CON group. After 6 h, pH values decreased significantly across all groups. Concurrently, pH values in the carbon source groups (INU and MCP) were significantly lower than in the CON. The H_MCP exhibited a significantly lower pH than the L_MCP. Residual carbohydrate levels showed distinct kinetic phases (Figure 2B). After 6 h, levels decreased significantly in both the L_MCP and INU groups, with a similar trend in H-MCP. A significant decrease occurred between 6 and 24 h in all groups, reflecting vigorous microbial utilisation of carbohydrates and accelerated fermentation during this phase. After 24 h, levels remained stable, which was consistent with the pH trends and indicated that MCP utilisation primarily occurred within the initial 24 h.

**Figure 3:**
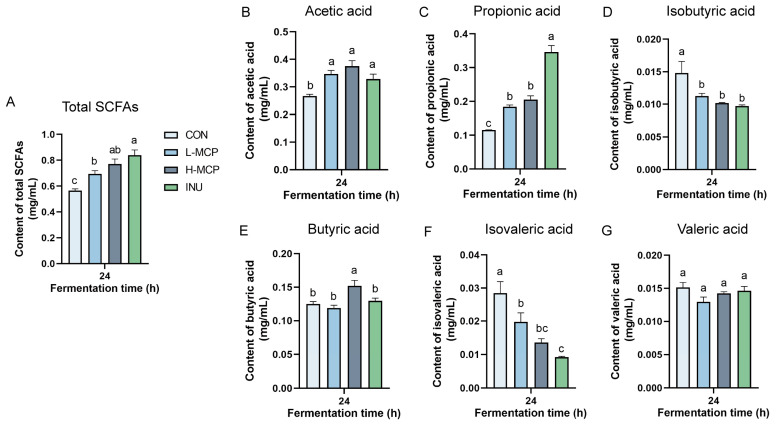
SCFAs content at 24 h: (**A**) Total SCFAs; (**B**) Acetic acid; (**C**) Propionic acid; (**D**) Isobutyric acid; (**E**) Butyric acid; (**F**) Isovaleric acid; (**G**) Valeric acid. Data represent mean ± SEM (*n* = 3). Lowercase letters denote significant differences between groups.

SCFA levels were measured in 24 h fermentation broths based on microbial utilisation of polysaccharides. A significant increase in total SCFA levels was observed in the L_MCP and H_MCP relative to the CON group (Figure 3A–G). In addition, total SCFAs in the H_MCP group exhibited no significant difference in comparison with the INU group. Compared with CON, both acetic acid and propionic acid levels were significantly elevated in the L_MCP and H_MCP. Acetic acid levels in the L_MCP and H_MCP (Figure 3B) showed no significant difference from the INU. Butyric acid was markedly increased in the H_MCP relative to the CON, INU, and L_MCP (Figure 3E). No significant intergroup variation was detected for valerate.

### 3.3. The Effect of MCP on Microbial Community Composition

**Figure 4:**
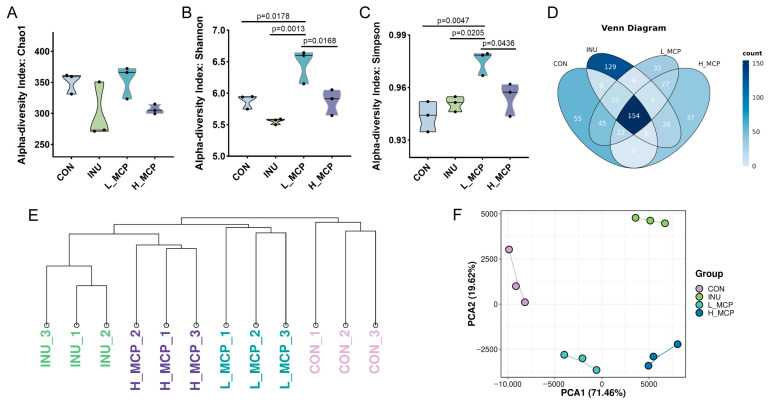
Microbial diversity analysis after 24 h fermentation. (**A**) Microbial richness estimated by Chao 1. (**B**) Microbial diversity measured by Shannon index. (**C**) Simpson index. (**D**) Venn diagram. (Venn diagram sample scale set: 0.7. If 70% of the samples within each group contain the ASV, the group is considered to contain the ASV.) (**E**) Cluster tree analysis of microbiota. (**F**) Cluster analysis of microbiota by PCA. (*n* = 3).

This study analysed the 16S rDNA gene sequences of microbial communities across all groups after 24 h of fermentation. Primarily, α-diversity was assessed. No marked variations in species richness were detected between the groups (Figure 4A–C). However, the microbial community diversity exhibited a significant increase in the L_MCP group in comparison to the other three groups. For beta-diversity, the Venn diagram (Figure 4D) revealed 154 ASVs shared among all groups. Cluster tree analysis showed distinct clustering of the four sample groups (Figure 4E), which was consistent with PCA results (Figure 4F) and confirmed pronounced differences in microbial composition between groups.

**Figure 5:**
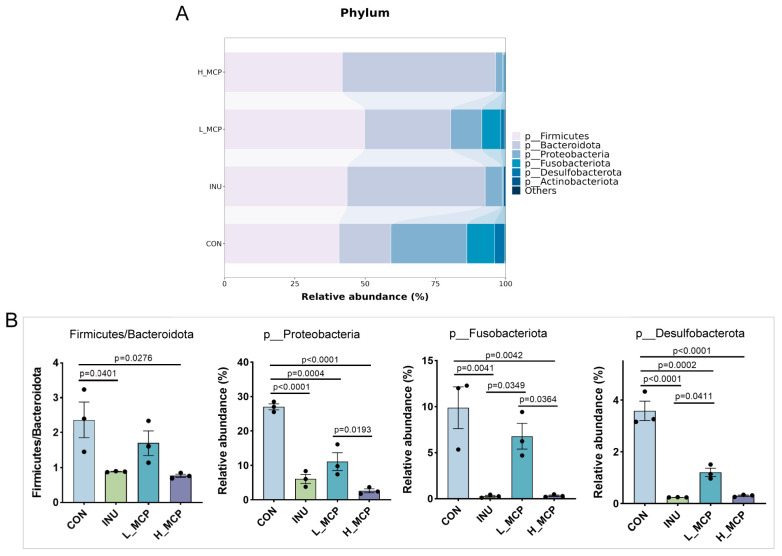
Phylum-level microbial composition after 24 h fermentation. (**A**) Stacked bar plot at the phylum level. (**B**) Relative abundance of phyla. Data represent mean ± SEM (*n* = 3).

At the phylum level (Figure 5A), the dominant taxa during MCP fermentation were Firmicutes, Bacteroidota, and Proteobacteria. The F/B ratio in the H_MCP was significantly lower than that in the CON (Figure 5B). The relative abundances of Proteobacteria and Fusobacteriota were significantly lower in the H_MCP in contrast to the CON and L_MCP.

**Figure 6:**
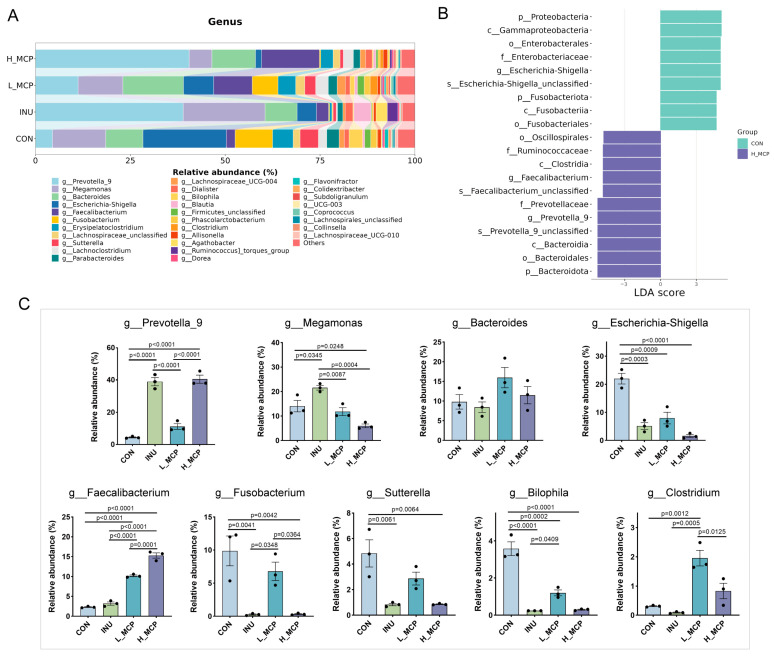
Genus-level microbial community structure after 24 h fermentation. (**A**) Stacked bar plot at the genus level. (**B**) Linear discriminant analysis (LDA). (**C**) Relative abundance of genera. Data represent mean ± SEM (*n* = 3).

A genus-level correlation matrix was constructed for the various samples (Figure S2). H_MCP group exhibited the weakest correlations with the CON group (below 0.39), while the strongest correlations were observed between H_MCP and INU groups (above 0.84). However, correlations between L_MCP and H_MCP ranged from 0.53 to 0.85, indicating that carbohydrate concentrations within the system influenced growth competition among different bacterial genera. To further investigate key genera metabolising MCP, the top 30 genera were analysed (Figure 6A–C). Linear discriminant analysis (LDA) revealed that *g_Prevotella_9* and *g_Faecalibacterium* significantly promoted the fermentation of H_MCP, occupying dominant positions within the gut microbiota. This finding suggested that MCP fermentation significantly enhanced the growth of beneficial bacteria. Furthermore, MCP reduced harmful bacterial abundance. The H_MCP group exhibited significantly lower levels of *g_Megamonas*, *g_Escherichia-Shigella*, *g_Fusobacterium*, *g_Sutterella*, and *g_Bilophila* in contrast to the CON. Notably, L_MCP group displayed markedly elevated levels of *g_Faecalibacterium* and *g_Clostridium*.

### 3.4. MCP-Induced Metabolic Changes During In Vitro Fermentation

**Figure 7:**
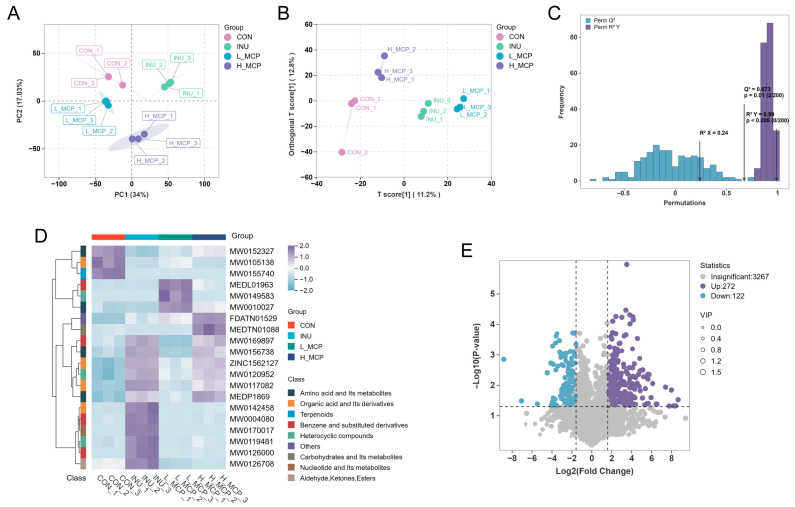
MCP-induced changes in microbial metabolism during in vitro fermentation. (**A**) PCA. (**B**) OPLS-DA analysis. (**C**) OPLS-DA verification diagram. (**D**) Visualization of differentially expressed metabolites via heatmap analysis (Table S2: Compound names corresponding to the substance index). (**E**) Volcanic map of H_MCP vs. CON.

The principal component analysis (PCA) was employed to analyse metabolites across groups (Figure 7A). The complete separation and clustering of groups indicated that differing carbohydrate categories in the fermentation broth were utilised to varying degrees by microorganisms, leading to significant alterations in the composition of generated metabolites. The orthogonal partial least squares discriminant analysis (OPLS-DA) model (Figure 7B) revealed a significant separation between the groups. In 200 random permutations (Figure 7C), R^2^Y = 0.99 (*p* < 0.005) and Q^2^ = 0.673 (*p* = 0.01) confirmed the reliability of the sample data and the model’s robust predictive capability.

Analysis of the top 20 significantly altered metabolites (Figure 7D) revealed that substances significantly enriched in the INU group included 2-Methoxyhexadecanoic acid, 3-Iodothyronamine, and Xanthine. The L_MCP group showed significant enrichment of Salicylamide, Ginkgolide C, and L-Valine. In contrast, the H_MCP group was enriched in Xanthopterin monohydrate, Lyxo-2-Hexulose, Taxol C, Ser-Glu-Lys-Ile-Asp, 10-Hydroxy-2-decenoic acid, Tetrahydropteridine, Indole-3-propionic acid, and His-Leu. Notably, some compounds significantly enriched in both the INU and H_MCP groups were identical, likely due to functional similarities between certain microbial populations enriched in both groups during fermentation.

To identify metabolites that differed significantly between the H_MCP and CON groups, the following thresholds were applied: fold change (FC) ≥ 3, *p*-value < 0.05, and variable importance in projection (VIP) > 1. As shown in Figure 7E, relative to the CON, 272 metabolites were enriched and 122 were depleted in the H_MCP group.

**Figure 8:**
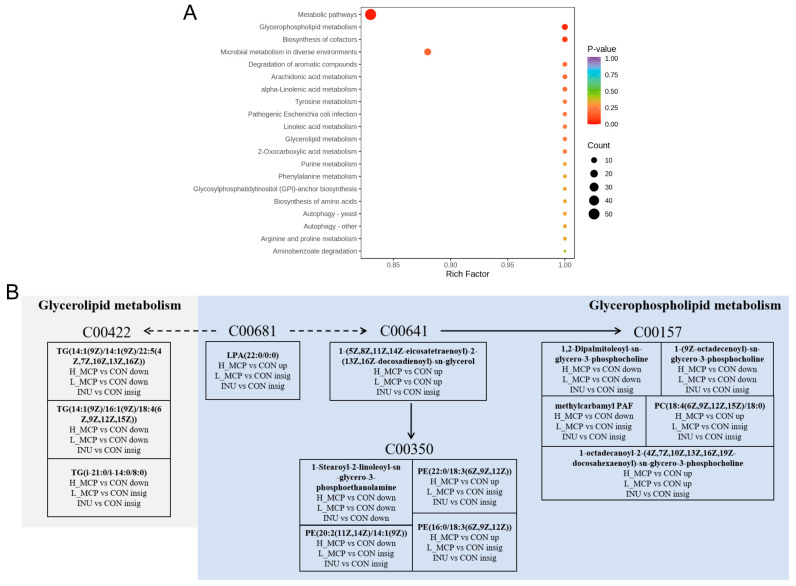
Microbial metabolic pathway analysis. (**A**) The enriched pathways in H_MCP fermentation. (**B**) Network diagrams of glycerophospholipid metabolism and glycerolipid metabolism based on KEGG. (Solid lines denote single-step reactions, whereas dotted lines indicate multi-step transformations.) *n* = 3.

These differential metabolites were subsequently mapped to KEGG pathways (Figure 8A,B). MCP fermentation influenced multiple subpathways within microbial lipid metabolism, including glycerophospholipid metabolism, arachidonic acid metabolism, alpha-linolenic acid metabolism, linoleic acid metabolism, glycerolipid metabolism, and glycosylphosphatidylinositol (GPI)-anchor biosynthesis. Among these, glycerophospholipid metabolism exhibited significant alterations. Following H_MCP fermentation, glycerophospholipid species LPA (22:0/0:0), PE (22:0/18:3 (6Z, 9Z, 12Z)), PE (16:0/18:3 (6Z, 9Z, 12Z)), PC (18:4 (6Z, 9Z, 12Z, 15Z)/18:0), and 1-octadecanoyl-2-(4Z, 7Z, 10Z, 13Z, 16Z, 19Z-docosahexaenoyl)-sn-glycero-3-phosphocholine levels were significantly increased, whereas 1-Stearoyl-2-linoleoyl-sn-glycero-3-phosphoethanolamine, PE (20:2 (11Z, 14Z)/14:1 (9Z)), 1,2-dipalmitoleoyl-sn-glycero-3-phosphocholine, 1-(9Z-octadecenoyl)-sn-glycero-3-phosphocholine, and methylcarbamyl PAF levels were significantly decreased. Furthermore, three glycerolipid species in glycerolipid metabolism (TG (14:1 (9Z)/14:1 (9Z)/22:5 (4Z, 7Z, 10Z, 13Z, 16Z)), TG (14:1 (9Z)/16:1 (9Z)/18:4 (6Z, 9Z, 12Z, 15Z)), and TG (i-21:0/i-14:0/8:0)) were significantly decreased.

### 3.5. The Hypolipidemic Effect of MCP on High-Fat-Induced Lipid Accumulation in C. elegans

**Figure 9:**
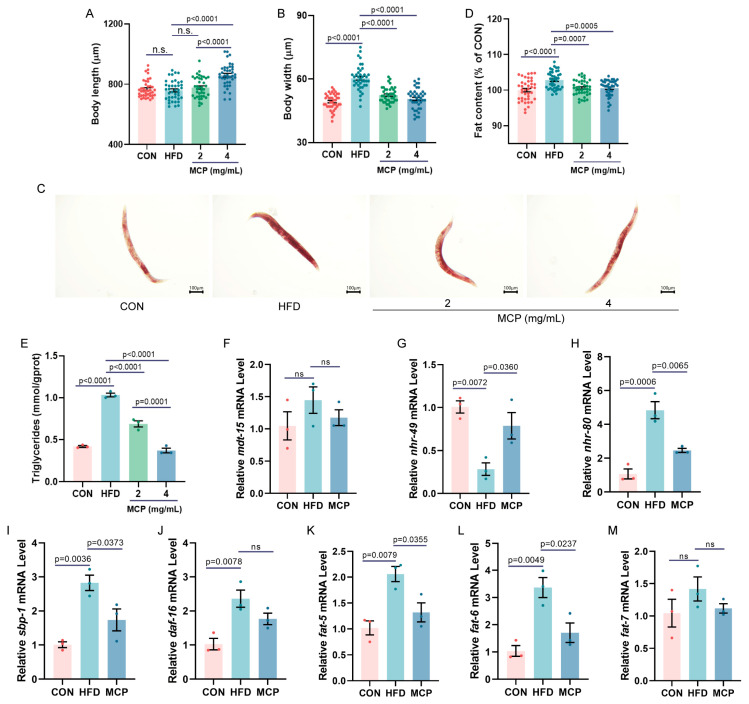
The lipid-lowering efficacy of MCP in alleviating high-fat-induced lipid accumulation model in *C. elegans*. (**A**) Quantification of body length. (**B**) Quantification of body width. (**C**,**D**) Fat deposition stained by Oil Red O and fat deposition quantified by Image J. Scale bar = 100 μm. Data represent mean ± SEM (*n* = 40). (**E**) Triglycerides. Effects of MCP on genes expression of *C. elegans*: (**F**) *mdt-15*; (**G**) *nhr-49*; (**H**) *nhr-80*; (**I**) *sbp-1*; (**J**) *daf-16*; (**K**) *fat-5*; (**L**) *fat-6*; (**M**) *fat-7*. Data represent mean ± SEM (*n* = 3).

To investigate the effects of MCP on host lipid metabolism, experiments were conducted using an HFD-induced lipid accumulation model in *C. elegans*. As shown in Figure 9A,B, the 4 mg/mL MCP group exhibited significantly increased body length and reduced body width compared to HFD group, demonstrating that MCP ameliorated the HFD-induced obesity phenotype. Lipid accumulation status was assessed via ORO staining (Figure 9C,D). In contrast to the HFD group, high- and low-dose MCP groups significantly reduced ORO levels, indicating that MCP intervention mitigated lipid accumulation in nematodes. Regarding oxidative stress in nematodes (Figure S3), no significant differences in ROS levels were observed between groups. Measurement of Triglycerides content revealed (Figure 9E) that both high- and low-dose MCP groups exhibited significantly lower Triglycerides levels than the HFD group, with the 4 mg/mL MCP group showing significantly lower Triglycerides levels than the 2 mg/mL MCP group. Given this dose-response, lipid metabolism-related gene expression analyses focused on the 4 mg/mL MCP group. As shown in Figure 9F–M, the intervention with MCP led to significantly higher expression of *nhr-49*, while *nhr-80*, *sbp-1*, and downstream genes *fat-5* and *fat-6* were markedly downregulated.

### 3.6. MCP-Mediated Lipid-Lowering Effect: Microbial Community Dependency Validation

**Figure 10:**
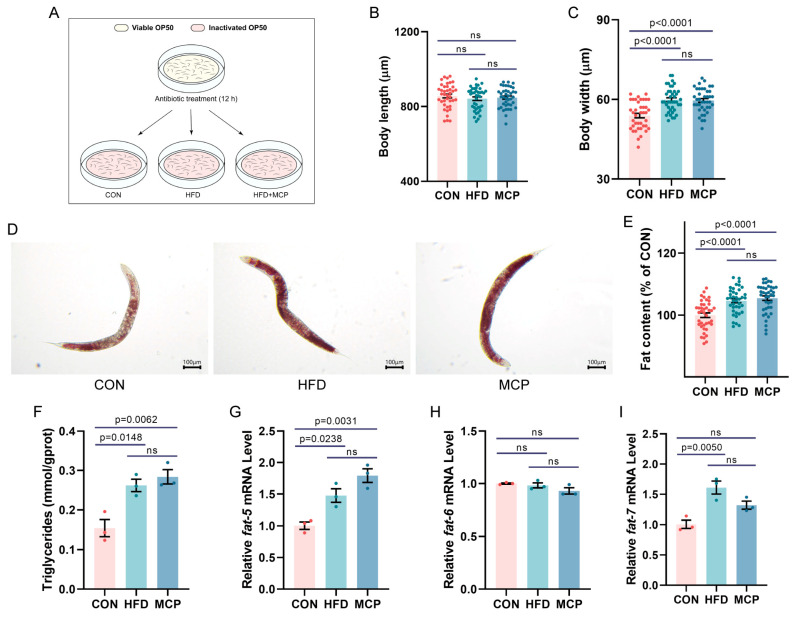
Failure analysis of lipid-lowering effect of MCP in the absence of flora. (**A**) Experimental scheme of antibiotic treatment in *C. elegans*. (**B**) Body length. (**C**) Body width. (**D**,**E**) Fat deposition stained by Oil Red O and fat deposition quantified by Image J. Scale bar = 100 μm. Data were expressed as mean ± SEM (*n* = 40). (**F**) Triglycerides. Effects of MCP on genes expression in antibiotic-treated *C. elegans*: (**G**) *fat-5*; (**H**) *fat-6*; (**I**) *fat-7*. Data represent mean ± SEM (*n* = 3).

Most dietary polysaccharides were degraded by gut microbiota, thereby regulating host metabolism. This study employed gentamicin treatment of *C. elegans* to simulate a microbiota depletion model, verifying whether MCP exerted its lipid-lowering effects via the microbial community. The experimental scheme for antibiotic treatment in *C. elegans* was illustrated in Figure 10A. The results demonstrated (Figure 10B–F) no significant differences in body length, body width, lipid deposition levels, or Triglycerides content between the HFD and MCP groups. Further assessment of lipid synthesis gene expression (Figure 10G–I) revealed that, compared to the CON group, *fat-5* expression was significantly upregulated in both the HFD and MCP groups. There were no significant variations in *fat-5* and *fat-7* gene expression between HFD and MCP groups. Notably, *fat-6* expression showed no significant variation across groups.

## 4. Discussion

Key findings revealed that MCP modulated microbial glycerophospholipid metabolic pathways during in vitro fermentation. The lipid-lowering effect of MCP from *M. crystallinum* L. was elucidated in this study. In *C. elegans*, MCP reduced lipid accumulation by regulating the *nhr-49*/*sbp-1* lipogenesis pathway. Furthermore, antibiotic-induced microbiota depletion abolished the lipid-lowering effect of MCP, suggesting a potential role of the gut microbiota in mediating MCP’s activity. This work established a scientific basis for the prospective use of halophyte polysaccharides as prebiotics.

Generally, the main bioactive components with lipid-lowering activity in plant extracts are considered to be polysaccharides, polyphenols, and flavonoids. In the present study, MCP was used at maximum concentrations of 5 mg/mL in the in vitro fermentation system and 4 mg/mL in the *C. elegans* culture medium. Consequently, the estimated concentrations of polyphenols derived from MCP were below 8.5 μg/mL and 6.8 μg/mL in the fermentation and *C. elegans* systems, respectively, while the estimated flavonoid concentrations were below 6.0 μg/mL and 4.8 μg/mL, respectively. These values were substantially lower than the reported effective doses [44,45]. Therefore, it is reasonable to attribute the activities observed in this study primarily to polysaccharides.

Previous work by M’Sakni et al. confirmed the presence of Gal-UA-rich polysaccharides in this plant, and the isolation and characterization of MCP in this study provided another clear example [21]. More importantly, this finding supported a broader consensus that the carbohydrate composition of halophytes was predominantly composed of uronic acid-rich polysaccharides [46]. Specifically, the high Gal-UA content (59.26%) in MCP not only conformed to this typical characteristic but also clearly classified it as a representative acidic polysaccharide from halophytes. The broad molecular weight distribution of MCP (PDI = 2.632) indicated its heterogeneous nature. Such structural complexity is common among plant-derived polysaccharides [47]. MCP remains suitable for preliminary bioactivity screening as a functional ingredient. Future studies aimed at fractionating MCP into more homogeneous fractions would help to establish clearer structure–activity relationships.

In vitro fermentation was employed as a primary system to evaluate the prebiotic properties of polysaccharides, with pH decline and substrate depletion serving as primary indicators of microbial activity [48]. In this study, both pH and residual MCP decreased markedly during fermentation, reaching a state of metabolic equilibrium by 24 h [49]. Previous studies demonstrated that indigestible carbohydrates were metabolized by the microbiota, yielding SCFAs [50]. It was also reported that acetate supported enterocyte energy and lipid-insulin signaling [51], while propionate modulated inflammation and glucose/cholesterol homeostasis [52]. Moreover, butyrate was shown to enhance energy expenditure and regulate intestinal lipid metabolism [53]. The results indicated that MCP served as a substrate for human fecal microbiota. These microbes significantly produced SCFAs (acetate, propionate, butyrate) through fermenting MCP, which were crucial for maintaining gut health. Mechanistically, gut bacteria utilize their CAZyme gene sets to degrade and ferment polysaccharides [54]. Specifically, *Prevotella* produced acetate by degrading carbohydrates, thereby regulating blood glucose and lipid levels [55,56]. *Faecalibacterium* generated butyrate by fermenting dietary fiber, promoting energy metabolism and inhibiting pathogen colonization [57]. Notably, MCP markedly elevated the levels of *g_Prevotella_9* and *g_Faecalibacterium* during in vitro fermentation. Concurrently, it inhibited the proliferation of potential pathogens (*g_Megamonas*, *g_Escherichia-Shigella*, *g_Fusobacterium*, *g_Sutterella*, and *g_Bilophila*) [22,58]. In the present in vitro fermentation system, MCP significantly altered microbial lipid-related metabolic pathways. Glycerophospholipid metabolism showed significant changes (*p* < 0.05), and some glycerolipid species in glycerolipid metabolism were significantly reduced. Glycerophospholipids and glycerolipids are two critical lipid classes: the former are vital components of biological membranes and key regulators of lipid metabolism, whereas the latter serve as the primary energy source, with elevated triglyceride levels being linked to fatty liver disease and cardiovascular disorders [59]. The observed changes in microbial lipid metabolism in the fermentation system raised the possibility that MCP might influence host lipid metabolism. This hypothesis was directly tested using an HFD-induced lipid accumulation model in *C. elegans*.

*C. elegans* was employed as a suitable model organism for lipid metabolism research because its metabolic genes are conserved in mammals [60]. The synthesis of monounsaturated fatty acids (MUFAs) was a critical step regulating lipid synthesis and degradation. In *C. elegans*, multiple nuclear receptors and transcription factors coordinately responded to lipid metabolic processes. *mdt-15* associated with the SREBP activation domain and exhibited specific binding to *nhr-49*. Both *nhr-49* and *nhr-80* participated in the regulation of fat utilization and fatty acid profiles [61]. *sbp-1* was a key transcription factor regulating fat metabolism, and *daf-16* was an important metabolic regulator. *C. elegans* encoded three Δ9 desaturases (*fat-5*, *fat-6*, and *fat-7*) [62]. In this context, MCP exerted a significant intervention effect on HFD-induced lipid accumulation in *C. elegans* by upregulating the expression of *nhr-49* and downregulating the expression of *nhr-80*, *sbp-1*, *fat-5*, and *fat-6*, thereby regulating lipid metabolism, reducing triglyceride levels, and alleviating fat deposition in a dose-dependent manner. Critically, MCP treatment did not significantly alter ROS levels relative to the HFD (Figure S3). Its lipid-lowering effect was not primarily mediated through ROS regulation, leading to a focus on the gut microbiota. *C. elegans* offered a unique experimental advantage for establishing microbiota-dependent causality: axenic individuals can be generated via bleaching and co-cultured with specific microorganisms, enabling definitive “on/off” comparisons unattainable in mammals [63]. Studies have demonstrated that different microorganisms distinctly regulate lipid metabolism in *C. elegans*—pathogenic bacteria typically enhance lipid accumulation, while probiotics exert lipid-lowering effects [64,65]. Leveraging this, this study employed antibiotic-induced microbiota depletion to directly test whether MCP’s lipid-lowering effect requires gut microbiota. Strikingly, under microbiota-depleted conditions, lipid-lowering effect of MCP disappeared, and lipid synthesis was primarily driven by *fat-5*. These findings confirmed the necessity of the gut microbiota for the bioactivity of MCP, while also revealing a compensatory shift in lipid metabolism upon microbiota loss. This mechanistic insight not only underscored the value of *C. elegans* as a high-throughput screening model for microbiota-targeted interventions but also established a critical foundation for future validation in mammalian systems.

## 5. Conclusions

This study investigated the biological effect by which MCP alleviated lipid accumulation using *C. elegans* models. Several points in this study warrant additional exploration in subsequent work. Initially, fractionation of MCP into more homogeneous components, along with detailed structural elucidation including glycosidic linkage analysis and NMR spectroscopy, is warranted to establish clearer structure–activity relationships. Additionally, future in vitro fermentation studies incorporating diverse donors would provide a more comprehensive understanding of inter-individual responses to MCP. Furthermore, HFD-induced lipid accumulation in *C. elegans* primarily reflects cellular lipid loading and steatosis, rather than the full physiological complexity of mammalian obesity. Therefore, future research should focus on validating its potential anti-obesity effects in mammalian models, such as HFD-induced obese mice, to confirm the translational relevance of these findings.

In conclusion, this work lays a foundation for further investigation into MCP as a novel prebiotic candidate, while highlighting the need for more detailed structural analysis, consideration of individual variability, and validation in mammalian models in future studies.

## Figures and Tables

**Table 1:** Calibration parameters for monosaccharide standards.

Standard	Standard Curve	R^2^
Fuc	y = 0.656x	0.9947
Rha	y = 0.4959x	0.9931
Ara	y = 0.8059x	0.9941
Gal	y = 0.7945x	0.995
Glc	y = 0.7571x	0.9955
Xyl	y = 0.6859x	0.9929
Man	y = 0.5633x	0.9952
Fru	y = 0.1379x	0.9965
Rib	y = 0.515x	0.998
Gal-UA	y = 0.3894x	0.9998
Gul-UA	y = 0.4583x	0.9993
Glc-UA	y = 0.6324x	0.9993
Man-UA	y = 0.3778x	0.9993

**Table 2:** The chemical composition and contents of MCP.

Chemical Composition	Neutral Sugar	Uronic Acid	Protein	Total Polyphenols	Total Flavonoids	Ash
%	16.28 ± 0.23	75.25 ± 3.89	2.03 ± 1.07	0.16 ± 0.01	0.08 ± 0.04	0.71 ± 0.16

## Data Availability

The original contributions presented in this study are included in the article/Supplementary Materials. Further inquiries can be directed to the corresponding author.

## Supplementary Materials

The following supporting information can be downloaded at https://www.mdpi.com/article/10.3390/foods15071153/s1. Additional data and materials that complement the main manuscript are available, including statistical analysis of microbiota sequencing and metabolomics data (Methods S1 and S2), quantitative measurements of spherical structures in a representative SEM image of MCP (Scale bar = 2 μm) (Figure S1), correlation analysis of samples based on genus-level data (Figure S2), ROS levels in *C. elegans* (Figure S3), the RT-qPCR primer sequences (Table S1) and the compounds name of differential metabolites (Table S2).

## Abbreviations

The following abbreviations are used in this manuscript:

*M. crystallinum* L.	*Mesembryanthemum crystallinum* L.
MCP	*Mesembryanthemum crystallinum* L. polysaccharides
HFD	High-fat diet
*C. elegans*	*Caenorhabditis elegans*
TG	Total triglycerides
Mw	Weight-average molecular weight
Mn	Number-average molecular weight
FTIR	Fourier Transform Infrared
SCFAs	Short-chain fatty acids
NGM	Nematode growth medium
L1	First-stage larvae
L4	Fourth-stage nematodes
OA	Oleic acid
PA	Palmitic acid
FuDR	5-fluoro-2′-deoxyuridine
H_2_DCF-DA	2,7-dichlorodihydrofluorescein-diacetate
KEGG	Kyoto encyclopedia of genes and genomes
PDI	Polydispersity index
UV	Ultraviolet
LDA	Linear discriminant analysis
PCA	Principal component analysis
OPLS-DA	Orthogonal partial least squares discriminant analysis
FC	Fold change
VIP	Variable importance in projection
ORO	Oil Red O
MUFAs	Monounsaturated fatty acids

## References

[1] World Obesity Federation (2024). World Obesity Atlas 2024.

[2] Ralston J.C., Lyons C.L., Kennedy E.B., Kirwan A.M., Roche H.M. (2017). Fatty Acids and NLRP3 Inflammasome-Mediated Inflammation in Metabolic Tissues. Annu. Rev. Nutr..

[3] Ge H., Ye X., Chen Q., Ye J., Chen J. (2023). Ligusticum chuanxiong prevents high-fat-diet-induced lipid metabolism disorder in mice by modulating the genes in the cholesterol pathway. Food Front..

[4] Wang M., Zhang B., Hu J., Nie S., Xiong T., Xie M. (2020). Intervention of five strains of Lactobacillus on obesity in mice induced by high-fat diet. J. Funct. Foods.

[5] Chooi Y.C., Ding C., Magkos F. (2019). The epidemiology of obesity. Metabolism.

[6] World Heart Federation (2025). Global levels and trends. World Heart Report 2025.

[7] Sharifi-Rad J., Quetglas-Llabrés M.M., Sureda A., Mardones L., Villagran M., Gürer E.S., Živković J., Ezzat S.M., Zayed A., Gümüşok S. (2024). Supercharging metabolic health with *Lycium barbarum* L.: A review of the therapeutic potential of this functional food for managing metabolic syndrome. Food Front..

[8] Lyu L., Fan Y., Bryrup T., Clos-Garcia M., Brix S., Eiken M., Stankevic E., Lund A.B., Knop F.K., Jørgensen N.R. (2025). Glucocorticoid-induced changes of the gut microbiota and metabolic markers in healthy young men: Outcome of a randomized controlled trial. Cell Rep. Med..

[9] Zhang L., Xu Z., Qin S., Liu R. (2025). Dietary Branched-Chain Amino Acids Restriction in High-Fat Diet-Induced Obese Mice: Effects on Metabolic Homeostasis, Adipose Inflammation, and Gut Microbiota. J. Nutr..

[10] Wang Z., Chen Y., Christian M., Dai X. (2025). Saikosaponin D ameliorates obesity and metabolic disorders via the gut microbiota-SCFAs-thermogenic fat axis. Food Biosci..

[11] Lin W., Wang X., Zhuang T., Wang Z., Yang L., Wang X., Ding L., Tao F. (2025). Lithospermum erythrorhizon polysaccharide alleviates obesity via gut microbiota-mediated reprogramming of bile acid and short-chain fatty acid metabolism. Int. J. Biol. Macromol..

[12] Lu X. (2023). Changes in the structure of polysaccharides under different extraction methods. eFood.

[13] Peng D., Cheng Y., Chen Y., Cai X., Bao H., Pan X., Ding W., Chen J., Li P. (2024). Chaihu Guizhi decoction ameliorates depression symptoms in chronic restraint stress-induced depressive rats by regulating gut microbiota and short-chain fatty acids. Food Saf. Health.

[14] Fang C.-Y., Chen S.-Y., Liao C.-C., Chen J.-D., Yen G.-C. (2025). Fagopyrum esculentum polysaccharides mitigate obesity by reshaping gut microbiota and enhancing lipid metabolism in high-fat diet-fed mice. Int. J. Biol. Macromol..

[15] Yuan D., Li C., Huang Q., Fu X., Dong H. (2023). Current advances in the anti-inflammatory effects and mechanisms of natural polysaccharides. Crit. Rev. Food Sci. Nutr..

[16] Wang J., Zhao Q., Liu H., Guo L., Ma C., Kang W. (2024). Regulating role of Pleurotus ostreatus insoluble dietary fiber in high fat diet induced obesity in rats based on proteomics and metabolomics analyses. Int. J. Biol. Macromol..

[17] Cebani S., Jimoh M.O., Sogoni A., Wilmot C.M., Laubscher C.P. (2024). Nutrients and phytochemical density in *Mesembryanthemum crystallinum* L. cultivated in growing media supplemented with dosages of nitrogen fertilizer. Saudi J. Biol. Sci..

[18] Bohnert H.J., Cushman J.C. (2000). The Ice Plant Cometh: Lessons in Abiotic Stress Tolerance. J. Plant Growth Regul..

[19] Hanen F., Riadh K., Samia O., Sylvain G., Christian M., Chedly A. (2009). Interspecific variability of antioxidant activities and phenolic composition in Mesembryanthemum genus. Food Chem. Toxicol..

[20] Kang Y.-W., Joo N.-M. (2023). Comparative Analysis on Phytochemical Properties, Anti-Oxidative, and Anti-Inflammatory Activities of the Different Organs of the Common Ice Plant *Mesembryanthemum crystallinum* L. Appl. Sci..

[21] M’sakni N.H., Majdoub H., Roudesli S., Picton L., Le Cerf D., Rihouey C., Morvan C. (2006). Composition, structure and solution properties of polysaccharides extracted from leaves of *Mesembryanthenum crystallinum*. Eur. Polym. J..

[22] Ye Z., Yu L., Zhang C., Gao Y., Zhao J., Narbad A., Chen W., Zhai Q., Tian F. (2024). Modulation of gut microbiota and metabolites by *Flammulina velutipes* polysaccharides during *in vitro* human fecal fermentation: Unveiling Bacteroides as a potential primary degrader. Food Chem..

[23] Song F., Ning F., Feng Y., Zhang Y., Gong F., Ning C., Yu Y., Zhang R., Han R., Qi Y. (2024). The polysaccharides from blackened jujube with ultrasonic assistance extraction: Optimization of extraction conditions, antioxidant activity and structural analysis. LWT.

[24] Liu Y., Li Y., Ke Y., Li C., Zhang Z., Wu Y., Hu B., Liu A., Luo Q., Wu W. (2021). In vitro saliva-gastrointestinal digestion and fecal fermentation of *Oudemansiella radicata* polysaccharides reveal its digestion profile and effect on the modulation of the gut microbiota. Carbohydr. Polym..

[25] Shi H., Wan Y., Li O., Zhang X., Xie M., Nie S., Yin J. (2020). Two-step hydrolysis method for monosaccharide composition analysis of natural polysaccharides rich in uronic acids. Food Hydrocoll..

[26] Bradford M.M. (1976). A rapid and sensitive method for the quantitation of microgram quantities of protein utilizing the principle of protein-dye binding. Anal. Biochem..

[27] Shen D., Kou X., Wu C., Fan G., Li T., Dou J., Wang H., Zhu J. (2021). Cocktail enzyme-assisted alkaline extraction and identification of jujube peel pigments. Food Chem..

[28] Mi S., Zhang X., Wang Y., Zheng M., Zhao J., Gong H., Wang X. (2022). Effect of different genotypes on the fruit volatile profiles, flavonoid composition and antioxidant activities of chilli peppers. Food Chem..

[29] Chen P., You Q., Li X., Chang Q., Zhang Y., Zheng B., Hu X., Zeng H. (2019). Polysaccharide fractions from Fortunella margarita affect proliferation of Bifidobacterium adolescentis ATCC 15703 and undergo structural changes following fermentation. Int. J. Biol. Macromol..

[30] Zhu M., Huang R., Wen P., Song Y., He B., Tan J., Hao H., Wang H. (2021). Structural characterization and immunological activity of pectin polysaccharide from kiwano (*Cucumis metuliferus*) peels. Carbohydr. Polym..

[31] Liu T., Nan M., Zhang S., Qin H., Zhao Z., Liu S., Mao J. (2024). Characterization of seselopsis tianschanica schischk polysaccharide (STSP) and its application in developing a functional fermented beverage with highland barle. Food Chem. X.

[32] Chen J., Liu L., Zhang Y., Jiao Y., Hou J., Liu F., Liu W. (2024). Characterization, antioxidant and antibacterial activity of neutral polysaccharides from oyster (*Crassostrea rivularis*). LWT.

[33] Lin P., Wang Q., Wang Q., Chen J., He L., Qin Z., Li S., Han J., Yao X., Yu Y. (2024). Evaluation of the anti-atherosclerotic effect for *Allium macrostemon* Bge. Polysaccharides and structural characterization of its a newly active fructan. Carbohydr. Polym..

[34] Impoco G., Carrato S., Caccamo M., Tuminello L., Licitra G. (2007). Quantitative analysis of cheese microstructure using SEM imagery. Commun. SIMAI Congr..

[35] Yang Q., Chang S.-L., Tian Y.-M., Li W., Ren J.-L. (2024). Glucan polysaccharides isolated from *Lactarius hatsudake* Tanaka mushroom: Structural characterization and in vitro bioactivities. Carbohydr. Polym..

[36] Zhang X., Aweya J.J., Huang Z.-X., Kang Z.-Y., Bai Z.-H., Li K.-H., He X.-T., Liu Y., Chen X.-Q., Cheong K.-L. (2020). In vitro fermentation of *Gracilaria lemaneiformis* sulfated polysaccharides and its agaro-oligosaccharides by human fecal inocula and its impact on microbiota. Carbohydr. Polym..

[37] Yu C., Ahmadi S., Shen S., Wu D., Xiao H., Ding T., Liu D., Ye X., Chen S. (2022). Structure and fermentation characteristics of five polysaccharides sequentially extracted from sugar beet pulp by different methods. Food Hydrocoll..

[38] Sun Y., Zhang S., He H., Chen H., Nie Q., Li S., Cheng J., Zhang B., Zheng Z., Pan S. (2023). Comprehensive evaluation of the prebiotic properties of *Dendrobium officinale* polysaccharides, β-glucan, and inulin during in vitro fermentation via multi-omics analysis. Int. J. Biol. Macromol..

[39] Liang X., Liu H., Wei Z., Ye G., Xu L., Ye Y., Qin J. (2023). Modulation of gut flore by dietary fibers from *Pyrus bretschneideri* Rehd.: Evaluation of fermentation characteristics using a colonic in vitro fermentation model. J. Funct. Foods.

[40] Su Q., Yang L., Xiao J., Cao H., Wang H. (2025). MYR ameliorated MSG-induced immune dysfunction in HFD mice via modulation of gut microbiota and SCFAs metabolism. Food Biosci..

[41] Logue J.B., Stedmon C.A., Kellerman A.M., Nielsen N.J., Andersson A.F., Laudon H., Lindström E.S., Kritzberg E.S. (2016). Experimental insights into the importance of aquatic bacterial community composition to the degradation of dissolved organic matter. ISME J..

[42] Liang L., Rasmussen M.-L.H., Piening B., Shen X., Chen S., Röst H., Snyder J.K., Tibshirani R., Skotte L., Lee N.C.Y. (2020). Metabolic Dynamics and Prediction of Gestational Age and Time to Delivery in Pregnant Women. Cell.

[43] Wan Q.-L., Meng X., Wang C., Dai W., Luo Z., Yin Z., Ju Z., Fu X., Yang J., Ye Q. (2022). Histone H3K4me3 modification is a transgenerational epigenetic signal for lipid metabolism in *Caenorhabditis elegans*. Nat. Commun..

[44] Dou J.-F., Wu C.-E., Fan G.-J., Li T.-T., Li X.-J., Zhou D.-D., Zhu J.-P., Li C.-M. (2023). Insights into the pigment and non-pigment phenolic profile of polyphenol extracts of jujube peel and their antioxidant and lipid-lowering activities. Food Biosci..

[45] Shen C.-Y., Wan L., Wang T.-X., Jiang J.-G. (2019). *Citrus aurantium* L. var. *amara* Engl. inhibited lipid accumulation in 3T3-L1 cells and *Caenorhabditis elegans* and prevented obesity in high-fat diet-fed mice. Pharmacol. Res..

[46] Mzoughi Z., Majdoub H. (2021). Pectic polysaccharides from edible halophytes: Insight on extraction processes, structural characterizations and immunomodulatory potentials. Int. J. Biol. Macromol..

[47] Wang Z., Zhou X., Shu Z., Zheng Y., Hu X., Zhang P., Huang H., Sheng L., Zhang P., Wang Q. (2023). Regulation strategy, bioactivity, and physical property of plant and microbial polysaccharides based on molecular weight. Int. J. Biol. Macromol..

[48] Li X., Peng B., Cheung P.C.-K., Wang J., Zheng X., You L. (2022). Depolymerized non-digestible sulfated algal polysaccharides produced by hydrothermal treatment with enhanced bacterial fermentation characteristics. Food Hydrocoll..

[49] Zang X., Jin X., Liu B., Zhang C., Narbad A., Zhao J., Chen W., Tian F., Yu L., Zhai Q. (2025). Morchella esculenta polysaccharides ameliorate obesity by enriching Bifidobacterium and Bacteroides. Food Biosci..

[50] Xie Z., Huang A., Cai J., Huang R., Chen M., Yu S., Zhang F., Zhu Z. (2024). Macro insights into the shared and distinct regulations of dietary polysaccharides on gut microbiota and their roles in obesity. Food Saf. Health.

[51] Kimura I., Inoue D., Hirano K., Tsujimoto G. (2014). The SCFA Receptor GPR43 and Energy Metabolism. Front. Endocrinol..

[52] Duscha A., Gisevius B., Hirschberg S., Yissachar N., Stangl G.I., Dawin E., Bader V., Haase S., Kaisler J., David C. (2020). Propionic Acid Shapes the Multiple Sclerosis Disease Course by an Immunomodulatory Mechanism. Cell.

[53] Li H., Yang S., Fan L., Luo L., Lei W., Tan P., Yue T., Gao Z. (2023). Investigating the contribution of mulberry leaf Fu tea to ameliorating metabolic disorders and remodeling gut microbiota in diabetic mice. Food Front..

[54] Bäumler A.J., Sperandio V. (2016). Interactions between the microbiota and pathogenic bacteria in the gut. Nature.

[55] Vandeputte D., Kathagen G., D’hoe K., Vieira-Silva S., Valles-Colomer M., Sabino J., Wang J., Tito R.Y., De Commer L., Darzi Y. (2017). Quantitative microbiome profiling links gut community variation to microbial load. Nature.

[56] Fan S., Ding Y., Hu Z., Zhang Z., Fu L., Zhang J., Zhu Y., Bai J., Xiao X. (2025). Inter-individual variation in human microbiota drives differential impacts on the fermentability of insoluble bran by soluble β-glucans from whole barley. Food Hydrocoll..

[57] Zou Y., Lin X., Xue W., Tuo L., Chen M.-S., Chen X.-H., Sun C.-H., Li F., Liu S.-W., Dai Y. (2021). Characterization and description of *Faecalibacterium butyricigenerans* sp. nov. and *F. longum* sp. nov., isolated from human faeces. Sci. Rep..

[58] Wu C., Yang F., Zhong H., Hong J., Lin H., Zong M., Ren H., Zhao S., Chen Y., Shi Z. (2024). Obesity-enriched gut microbe degrades myo-inositol and promotes lipid absorption. Cell Host Microbe.

[59] Wu G., Wang M., Du Z., Li Z., Han T., Xie Z., Gu W. (2025). Tea polyphenol EGCG enhances the improvements of calorie restriction on hepatic steatosis and obesity while reducing its adverse outcomes in obese rats. Phytomedicine.

[60] Yue Y., Li S., Shen P., Park Y. (2021). *Caenorhabditis elegans* as a model for obesity research. Curr. Res. Food Sci..

[61] Magner D.B., Wollam J., Shen Y., Hoppe C., Li D., Latza C., Rottiers V., Hutter H., Antebi A. (2013). The NHR-8 Nuclear Receptor Regulates Cholesterol and Bile Acid Homeostasis in *C. elegans*. Cell Metab..

[62] Brock T.J., Browse J., Watts J.L. (2007). Fatty acid desaturation and the regulation of adiposity in *Caenorhabditis elegans*. Genetics.

[63] Wang J., Lin Q., Liang Y. (2026). Unlocking the secrets of gut microbiota: The untapped potential of *Caenorhabditis elegans*. J. Adv. Res..

[64] Xie K., Liu Y., Li X., Zhang H., Zhang S., Mak H.Y., Liu P. (2022). Dietary *S. maltophilia* induces supersized lipid droplets by enhancing lipogenesis and ER-LD contacts in *C. elegans*. Gut Microbes.

[65] Gu M., Werlinger P., Cho J.-H., Jang N., Choi S.S., Suh J.-W., Cheng J. (2023). *Lactobacillus pentosus* MJM60383 inhibits lipid accumulation in *Caenorhabditis elegans* induced by *Enterobacter cloacae* and glucose. Int. J. Mol. Sci..

