# Supplementary material for: Physicochemical Characterization, Prebiotic Potential, and Lipid-Lowering Effect of Mesembryanthemum crystallinum L. Polysaccharide
Source: Foods. 2026 Mar 27;15(7):1153. doi: 10.3390/foods15071153 (PMC13073345; doi:10.3390/foods15071153)
Supplement: Supplementary file 1 [file foods-15-01153-s001.zip › foods-4198841-SI.pdf]

Supplementary Materials

Methods S1. Statistical analysis of microbiota sequencing data.

Methods S2. Statistical analysis of metabolomics data.

Figure S1. Quantitative measurements of spherical structures in a representative SEM image of MCP.

Figure S2. Correlation analysis of samples based on genus-level data.

Figure S3. ROS levels in *C. elegans*.

Table S1. The RT-qPCR primer sequences (*C. elegans*).

Table S2. The compounds name of differential metabolites.

Methods S1. Statistical analysis of microbiota sequencing data.

For microbiota sequencing data, raw sequencing data were subjected to quality control and preliminary filtration using Cutadapt 1.9, FLASH 1.2.8, Fqtrim, and Vsearch 2.3.4. Denoising was performed using the DADA2 plugin (clustered at 100% similarity) within the QIIME 2 platform to generate amplicon sequence variant (ASV) feature tables and representative sequences. To identify differentially abundant taxa between two groups, the Wilcoxon rank-sum test was performed. To account for the issue of multiple testing inherent in analyzing hundreds of microbial taxa simultaneously, the raw p-values were adjusted using the Benjamini–Hochberg false discovery rate (FDR) correction. Taxa with an adjusted p-value (q-value) < 0.05 were

considered statistically significant. Subsequent statistical analysis and data visualization were conducted using the Omicstudio online data analysis platform (https://www.omicstudio.cn/home).

Methods S2. Statistical analysis of metabolomics data.

For non-targeted metabolomics, raw mass spectrometry data were converted to mzML format using ProteoWizard. Peak extraction, alignment, and retention time correction were performed using the XCMS programme. Peaks with a missing rate exceeding 50% across all sample groups were filtered. Blank values were imputed using KNN filling supplemented by the 1/5 minimum value (where blank values > 50% employed the 1/5 minimum value imputation, and blank values < 50% utilised KNN filling). Multiple testing correction was performed using the Benjamini–Hochberg (BH) procedure. Differential metabolites were identified using the following criteria: For two-group analysis, differential metabolites were determined by VIP (VIP > 1) and adjusted P-value (q-value < 0.05, Student's t test). For multi-group analysis, differential metabolites were determined by VIP (VIP > 1) and adjusted P-value (q-value < 0.05, ANOVA). VIP values were extracted from OPLS-DA results, which also contain score plots and permutation plots generated using R package MetaboAnalystR. The data were log-transformed (Log2) and mean-centered before OPLS-DA. In order to avoid overfitting, a permutation test (200 permutations) was performed. Statistical analysis and

data visualization were performed using the Metware Cloud online data analysis platform (https://cloud.metware.cn/). Metabolic enrichment and pathway analysis traced biochemical pathways of differentially expressed metabolites (KEGG, http://www.genome.jp/kegg/).

Figure S1

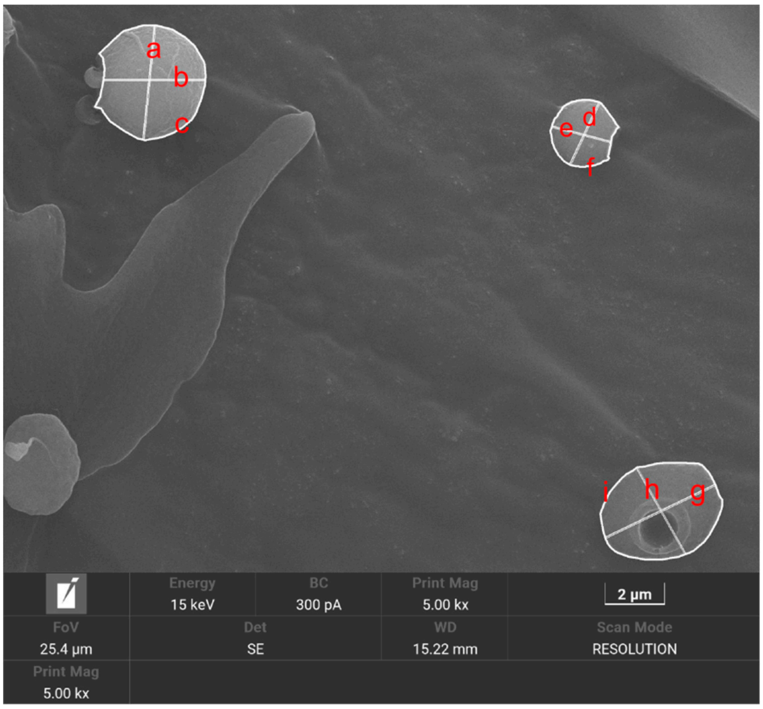

Figure S1. Quantitative measurements of spherical structures in a representative SEM image of MCP. Scale bar = 2 μm. Particle diameter (a, b, d, e, g, h) and perimeter (c, f, i) were analyzed using ImageJ 1.8.0 processing software. The measured values (μm) are: a = 4; b = 3; c = 12; d = 2; e = 2; f = 7; g = 4; h = 3; i = 12.

Figure S2

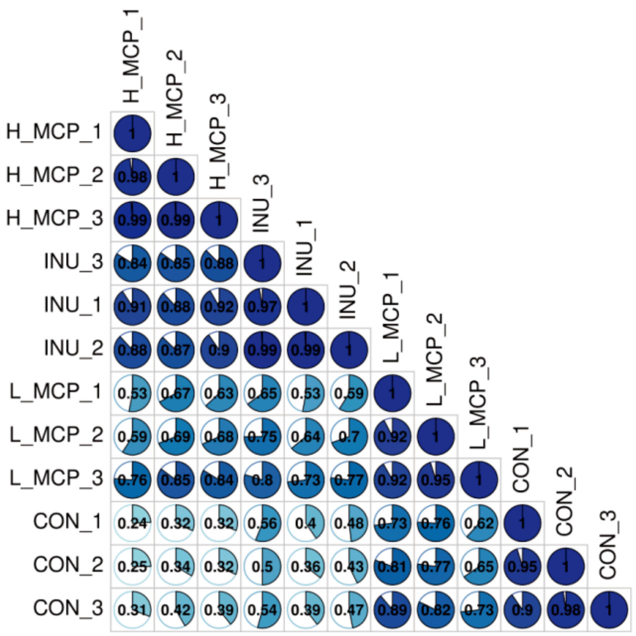

Figure S2. Correlation analysis of samples based on genus-level data.

Figure S3

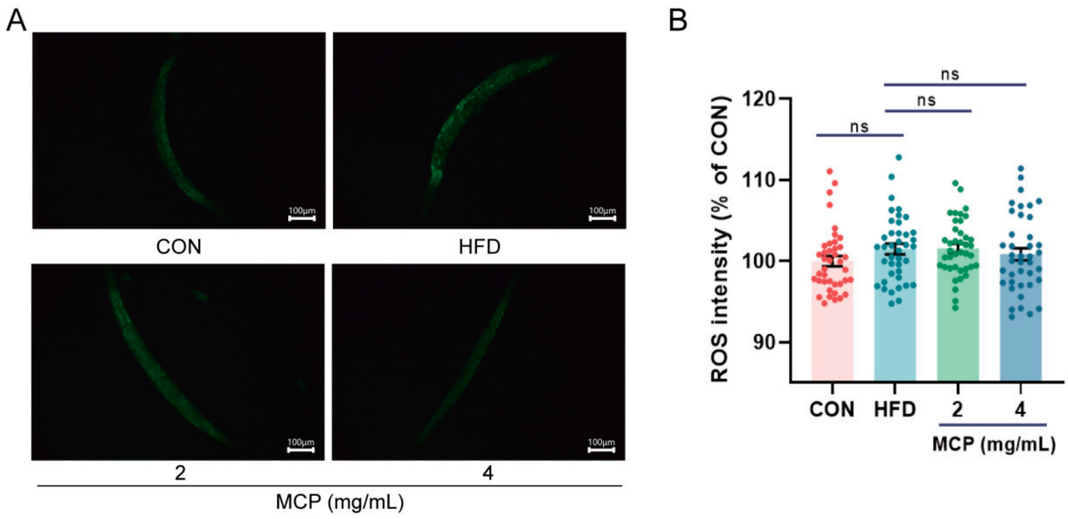

Figure S3. ROS levels in *C. elegans*. (A, B) Typical fluorescence photograph of ROS content and ROS content quantified by Image J. Scale bar = 100 μm. Data were expressed as mean ± SEM (n = 40).

Table S1. The RT-qPCR primer sequences (*C. elegans*).

Primer sequences used for RT-qPCR (5′→3′)	
Primer name	Sequences(5'-3')
act-1	F:CCATCATGAAGTGCGACATTG
act-1	R:CATGGTTGATGGGGCAGGAG
mdt-15	F:CAGTGCCTTCCGTGTTAC
mdt-15	R:GTTGATGCCTGTGTTGTTG
daf-16	F:TCGTCGTCTCGTGTTTCTCCA
daf-16	R:TTCCATAGGCACCCGGTAGTG
nhr-49	F:GTCGTTATTGTCGCTTTCAA
nhr-49	R:TCCGACACCGTTGCTGTTTC
nhr-80	F:TGAGGTTCAGGAGCCAAATAG
nhr-80	R:GAAGGAGGTGGACGATGAGA
sbp-1	F:CATGAATTCATTCGAGGGAGACGTCCC
sbp-1	R:CATGAATTCCTGATGTGGAGTCATCGC
fat-5	F:GGGCTACAGTTGGATGGGTATT
fat-5	R:CGGGTCAGCATCAGTATCCG
fat-6	F:AAGATTGAGAAGGACGGCGG
fat-6	R:TCACGGTTTGCCATTTTGCC
fat-7	F:AAGGAGCATGGAGGCAAACT
fat-7	R:TTCTCAACGGCGGAAACAGA

Table S2. The compounds name of differential metabolites.

Index	Compounds
MW0152327	Leu-Glu-Pro-His-Ile
MW0105138	3-Sulfinylpyruvic acid
MW0155740	{(1r,2r,3r)-2-[(3e)-4,8-Dimethylnona-3,7-Dien-1-Yl]-2-Methyl-3-[(1e,5e)-2,6,10-Trimethylundeca-1,5,9-Trien-1-Yl]cyclopropyl}methyl Trihydrogen Diphosphate
MEDL01963	Salicylamide
MW0149583	Ginkgolide C
MW0010027	L-Valine
FDATN01529	Xanthopterin monohydrate
MEDTN01088	Lyxo-2-Hexulose
MW0169897	Taxol C
MW0156738	Ser-Glu-Lys-Ile-Asp
ZINC1562127	10-Hydroxy-2-decenoic acid
MW0120952	Tetrahydropteridine
MW0117082	Indole-3-propionic acid
MEDP1869	His-Leu
MW0142458	2-Methoxyhexadecanoic acid
MW0004080	3-Iodothyronamine
MW0170017	Xanthine
MW0119481	3-Bromo-7-nitroindazole
MW0126000	Oxibendazole
MW0126708	Tert-butyl 5-methyl-6-oxo-5,6-dihydro-4h-imidazo[1,5-a]thieno[2,3-f][1,4]diazepine-3-carboxylate
